# Experimental control system of the X-ray magnetic circular dichroism endstation at Hefei Light Source-II

**DOI:** 10.1107/S160057752501135X

**Published:** 2026-01-22

**Authors:** Anbo Kong, Liuguo Chen, Kai Chen, DaDi Zhang, Xiaokang Sun, Gongfa Liu

**Affiliations:** a National Synchrotron Radiation Laboratory University of Science and Technology of China Hefei Anhui230029 People’s Republic of China; University College London, United Kingdom

**Keywords:** *Bluesky*, data acquisition, *EPICS*, experimental control, fly scan

## Abstract

The Hefei Light Source-II (HLS-II) facility is undergoing upgrades to its beamline and endstation to enhance its synchrotron radiation capabilities. For the X-ray Magnetic Circular Dichroism endstation, an experimental control system leveraging *Bluesky* and *Experimental Physics and Industrial Control System* has been developed. This control system implements three distinct scan modes: step scan, fly scan and soft fly scan. The system architecture employs *Queue Server* for automated experiment orchestration and *Ophyd* based device abstraction, enabling seamless adaptation across multiple HLS-II beamlines. During software development, three key system features are included: (1) a simulation debugging environment combining a virtual server platform, virtual devices and virtual input/output controllers for closed-loop testing; (2) unified control interfaces through component abstraction; and (3) real-time data acquisition with noise-reduction methods. The implemented soft fly scan mode demonstrates particular advantages in experiments, achieving a tenfold reduction in measurement duration while maintaining signal-to-noise ratio levels comparable to conventional step scan, significantly enhancing both experimental efficiency and data quality for the user.

## Abstract 2

An Experimental Control System leveraging *Bluesky* and *EPICS* has been developed for the X-ray Magnetic Circular Dichroism endstation at Hefei Light Source-II. The design of a simulation debugging environment and the performance of three distinct scan modes have also been discussed.

## 1. Introduction

The Hefei Light Source-II (HLS-II) is a synchrotron radiation facility in China with an electron ring energy of 0.8 GeV, spectrally strongest in the VUV and soft X-ray (Li *et al.*, 2007 ▸). The HLS-II X-ray Magnetic Circular Dichroism (XMCD) beamline contains two branches: one is the general soft X-ray absorption spectroscopy (XAS) endstation and *in situ* XAS endstation, and the other is the soft XMCD endstation. The XMCD endstation provides experimental methods such as XAS, XMCD and X-ray magnetic linear dichroism. These methods are based on the synchrotron radiation source with high brightness, high energy resolution and tunable polarization. Compared with conventional magnetic measurement technologies, these methods are ultrahigh surface/interface sensitive and element-specific (Van Der Laan, 2013 ▸). They can determine a sample’s atomic coordination environment and electronic structure, measure ferromagnetic and antiferromagnetic order simultaneously, and distinguish between orbital and spin magnetic moments. The XMCD endstation enables quantitative analysis on magnetic moment size and direction, magnetocrystalline anisotropy and crystal field of magnetic materials. Its capabilities have been widely used in energy materials, magnetic materials, ferroelectric materials, and superlattice materials and other material fields.

The HLS-II control system is a distributed control system based on *Experimental Physics and Industrial Control System* (*EPICS*). The experimental control and data acquisition software of each endstation was developed independently without a unified architecture (Zhang & Liu, 2021 ▸). With the upgrading and renovation of the beamline endstations, the experimental control system (ECS) design concept needs to shift from the traditional equipment control-centered to the scientific experiment-centered. The ECS aims to achieve automation and intelligence of experimental control and scientific data acquisition. While synchrotron radiation facilities around the world are being upgraded, many facilities are developing the next generation of synchrotron radiation beamline control systems. Based on two different underlying control architectures, *EPICS* and *TANGO*, upper-level experimental control software frameworks and application systems such as *GDA* (Gibbons *et al.*, 2011 ▸), *Sardana* (Reszela *et al.*, 2014 ▸), *Bluesky* (Allan *et al.*, 2019 ▸) and *BLISS* (Guijarro *et al.*, 2023 ▸) have been developed. *Bluesky*, based on *EPICS* and developed by the National Synchrotron Light Source-II (NSLS-II), is a collection of open-source Python libraries for scientific data acquisition, management and analysis. These *Bluesky* components meet the requirements of the HLS-II ECS. The XMCD endstation employs *Bluesky* and *EPICS* as its ECS framework, and uniformly abstracts the controlled devices and experimental procedures required for various scan modes, including step scan, fly scan and soft fly scan. The graphical user interface (GUI) is developed in a modular manner to implement functions such as experimental procedure control and data display. A unified automated data acquisition system is established to create a standard data storage format, forming an ecosystem for the life cycle management of experimental data such as experimental control, data acquisition and online analysis.

In spectral measurements, it is necessary to take data over a range of photon energies. During step scan, the motor gradually moves to each energy position, stops at each point and the detector readings are then collected. This process requires the motor to undergo multiple cycles of deceleration, stopping and acceleration. The dead time caused by the motor movement constitutes a significant portion of the total completion time, leading to a long scan process. One solution that effectively reduces scan time is to implement fly scan, which enables the motor to move continuously during the scan process while synchronously collecting detector data. Some endstations at global synchrotron radiation facilities have already adopted this fast continuous scan system, including the hardware fly scan (Hidas *et al.*, 2022 ▸; Reszela *et al.*, 2013 ▸) that uses hardware to trigger the detector and software fly scan (Alzubi *et al.*, 2022 ▸; Lin *et al.*, 2013 ▸) which relies on software synchronization. The HLS-II XMCD endstation offers both hardware fly scan and software fly scan modes. These modes not only shorten the scan time but also improve the experimental efficiency while maintaining the consistency of the spectrum.

Simulation of a beamline endstation in modern synchrotron radiation facilities represents a crucial aspect of their design and debugging processes. Simulation experiments of the ECS can be applied to parameter optimization and control system development and testing (Smith *et al.*, 2021 ▸; Campbell *et al.*, 2021 ▸; Lukaszewski & Klys, 2023 ▸). At present, the studies on beamline endstation virtualization primarily focus on developing simulation tools and integrating these tools with ECSs to optimize beamline parameters through simulation experiments. There is a lack of research concerning the simulation of device functions and behaviors from a control perspective based on communication protocols. During the development of the XMCD ECS, virtualization technologies, including server platform virtualization and device virtualization, are used to establish a simulation debugging environment. This approach can reduce hardware dependence, enhance the efficiency of control software development and testing, and provide support for control system automation deployment.

## 2. Design of the ECS

### 2.1. Hardware architecture

**Figure 1:**
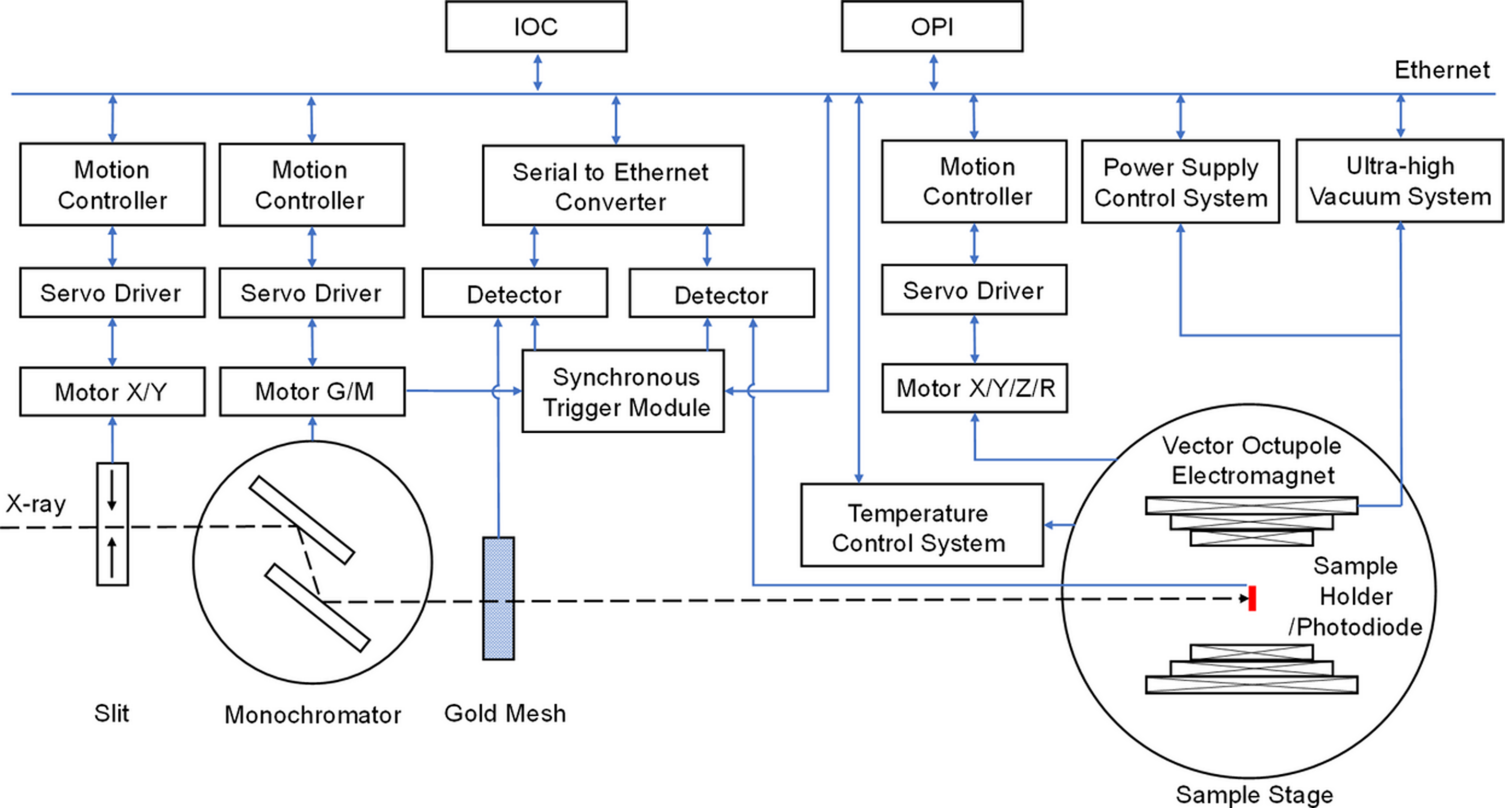
Hardware architecture of the XMCD ECS.

The hardware architecture of the XMCD ECS consists of two subsystems: the motion control system and the data acquisition system, as shown in Fig. 1 ▸. The motion control system comprises motors, servo drivers and motion controllers that regulate the movements of the slit, monochromator and sample stage. The data acquisition system includes the detectors and synchronous trigger module, the vector octupole electromagnet power supply control system and ultra-high vacuum system, and the sample stage temperature control system. The motion control system and the data acquisition system communicate with the input/output controller (IOC) and operator interface via Ethernet.

The AM23SS2DGA-N motors of the slit and monochromator use the EtherCAT bus protocol, and are controlled by the ZMC464 motion controllers and the SSDC06-ECX-H servo drivers, enabling high-precision multi-axis synchronous motion. During fly scan (Zhang *et al.*, 2024 ▸), the monochromator motion controller manages the synchronous motion of multi-axis motors according to the trajectory. The position encoder signals are transmitted to the synchronous trigger module which calculates the motor positions based on the encoder signals, generates trigger signals and triggers the detectors to collect data.

The XMCD endstation has three detection methods: total electron yield (TEY), transmission and photoluminescence (PL) (Goering *et al.*, 2001 ▸). The incident photon beam intensity is collected through the gold mesh positioned along the optical path in front of the sample. The photon beam intensity absorbed by the sample is indirectly measured by TEY method. In TEY mode, the sample is grounded by an electrometer to measure the sample current. In transmission mode, the photon beam intensity after the sample is detected directly by a photodiode and measured by an electrometer. The PL signal is also detected by a photodiode. The gold mesh signal and sample signal are all collected by Keithley 6517B electrometers and connected to the Ethernet through a serial-to-Ethernet converter. The synchronous trigger module is based on the Xilinx ZYNQ7020 platform. By decoding the encoder signals, it generates accurate trigger signals to synchronize the data acquisition of the electrometers.

The XMCD endstation employs the American ADC’s R2D2-100 vector octupole electromagnet vacuum chamber system, which is capable of achieving a maximum magnetic flux density of 1 T at the sample position, and can adjust the magnetic field direction arbitrarily. The power supply control system is designed to manage the power supply, control and interlocking of the electromagnet. The XMCD endstation uses a customized four-dimensional liquid helium low-temperature sample stage provided by Fermion. This sample stage consists of a low-temperature weak current measurement sample rod and a four-dimensional translation/rotation stage. All components of the ultra-low temperature sample stage are made of non-magnetic, low-temperature resistant and ultra-high vacuum compatible special materials to ensure optimal thermal and mechanical properties at extremely low temperatures. The sample temperature range is 10–300 K.

### 2.2. Software architecture

**Figure 2:**
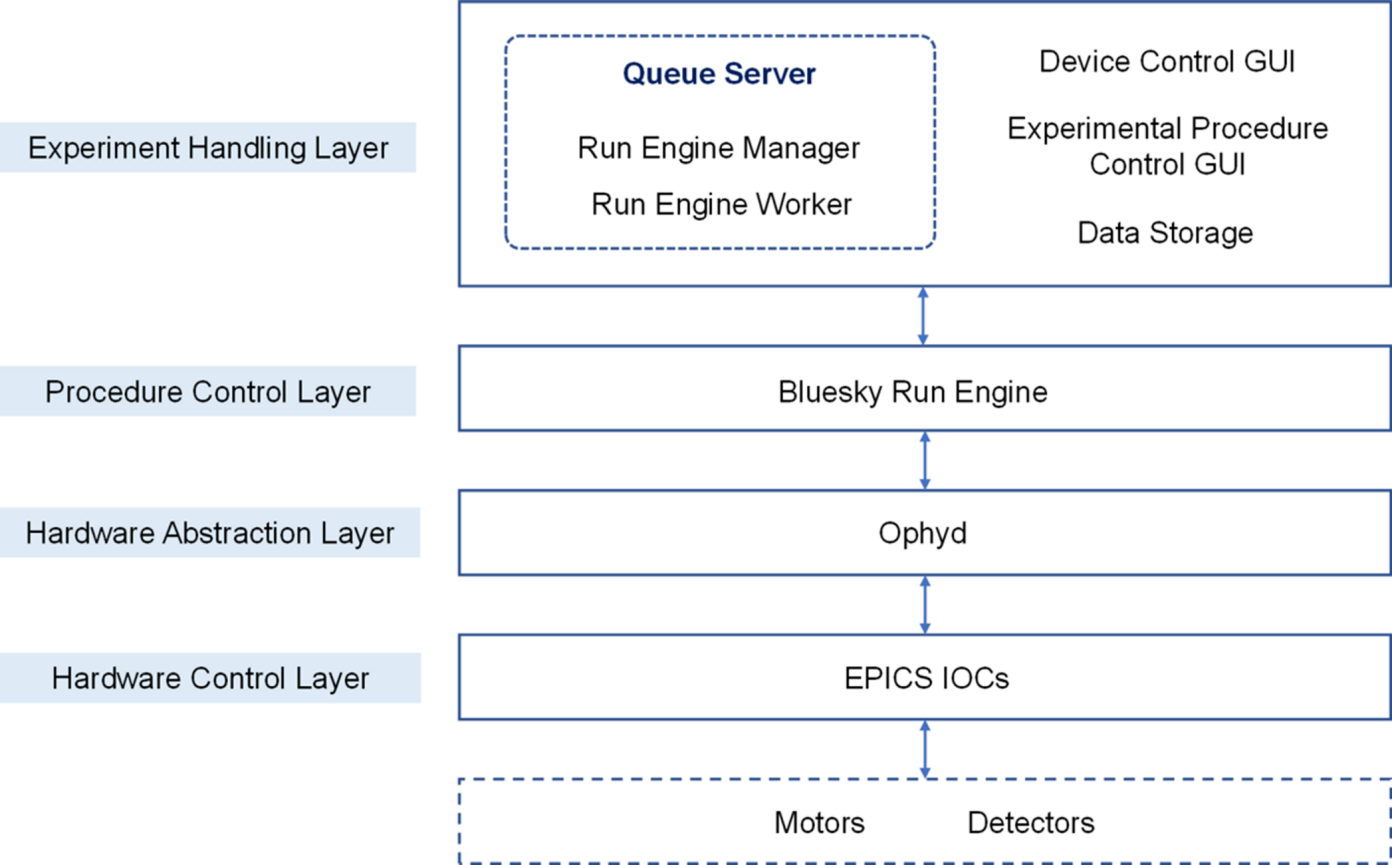
Software architecture of the XMCD ECS.

The software architecture of the XMCD ECS is organized into a hierarchical structure comprising four layers: the hardware control layer, hardware abstraction layer, procedure control layer and experiment handling layer, as shown in Fig. 2 ▸. The hardware control layer is based on *EPICS*, and the hardware abstraction layer, procedure control layer and experiment handling layer are developed based on *Bluesky*. The vector octupole electromagnet power supply control system and ultra-high vacuum system, and the sample stage temperature control system each have their own dedicated software modules.

The hardware control layer is a distributed architecture based on *EPICS*. The IOC applications include the detector IOC, the motor IOC and the energy-position conversion IOC. These IOCs collectively provide the process variables (PVs) essential for the hardware abstraction layer. The hardware abstraction layer uses *Ophyd* from *Bluesky* to convert PVs into Python objects. The procedure control layer executes *Bluesky* ‘Plans’, such as step scan, and associates various metadata with experimental raw data. It provides an interface to the experiment handling layer in the form of scripts. The experiment handling layer is primarily utilized for organizing and managing Plans. Additionally, it is responsible for collecting and processing the data generated during these experiments.

*Bluesky Queue Server* (Rakitin *et al.*, 2022 ▸) is the core of the XMCD ECS. Users upload Plans to *Queue Server*, and Plans are executed autonomously in sequence. *Queue Server* runs as a multi-process service with *Run Engine Manager* as the central component, responsible for maintaining and executing the Plan queue. Plans are executed in a separate process within the *Run Engine Worker* environment. *Queue Server* provides Plan queue management services for maintaining a queue of multiple Plans in *Redis*. The GUI is developed using the *Bluesky Queue Server* API (NSRL-II, 2025*a* ▸) and *Bluesky Widgets* (NSLS-II, 2025*b* ▸). A control architecture that separates control flow and data flow is adopted. This architecture ensures that critical tasks such as experimental procedure control and data acquisition are decoupled from secondary tasks like displaying real-time data on the client. This approach enhances the stability of the experimental procedure.

#### 2.2.1. Device and procedure abstraction

*Ophyd* abstracts experimental device PVs into Python objects with methods such as read() and set(). Motor and detector PVs are abstracted into *Ophyd Signals*, while multiple Signals are grouped to *Ophyd Device*, which can be configured and used as a unit. The gold mesh signal (*I*_0_) and sample signal (*I*_s_) are abstracted into *EpicsSignals*. The monochromator motor (motor G and motor M) signals are abstracted and combined into *EpicsMotors*. The position-energy conversion signal and energy-position conversion signal are abstracted into *EpicsSignals*. Signals are used to record the average values of the detector signals (*I*_0_mean_ and *I*_s_mean_) and their corresponding ratio (*I*_s_/*I*_0_).

The experimental procedure is encoded as Plan, which utilizes the Signals and Devices to execute a sequence of atomic instructions according to the experimental procedure in order to complete the entire experiment. *Bluesky* offers a variety of predefined Plans which are constructed from Stub Plans. When the built-in Plans do not fulfill the specific requirements of the endstation, Stub Plans can be used to customize a new Plan. The Plans supported by the XMCD endstation are count, set energy, step scan, fly scan and soft-fly scan.

**Figure 3:**
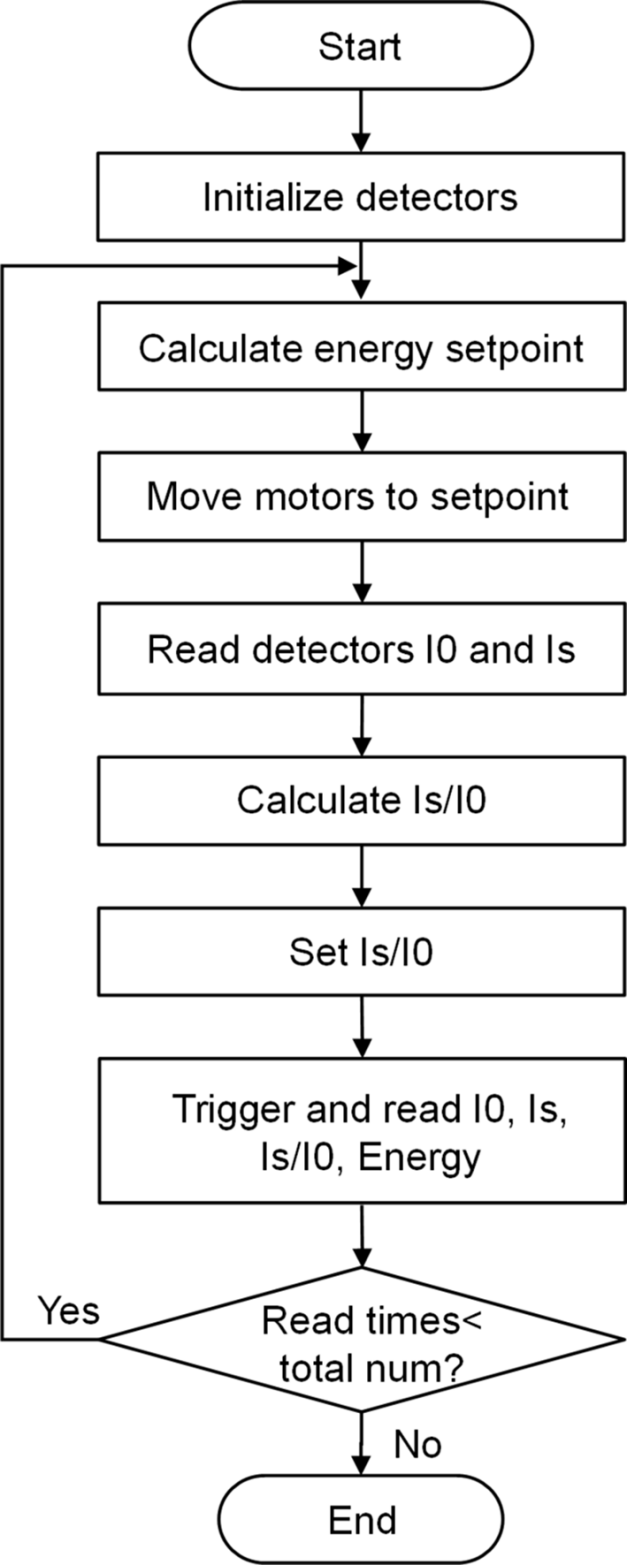
Step scan procedure.

The step scan procedure abstraction requires the definition of the input parameters, including the start and end energy, the scan step. The step scan procedure is shown in Fig. 3 ▸. The detector parameters such as filter and integration time are initialized before scanning. During the scan, if the current number of acquisition points does not reach the total number, the energy setpoint is calculated, and the motors are then moved to the positions corresponding to the energy setpoint. The detector data are recorded in one ‘Event’. This cycle is repeated until the entire procedure is completed.

*Bluesky* defines the fly scan through three steps: kickoff, complete and collect. A Flyer class is developed that incorporates these three methods. Fly scan is designed as a hardware fly scan Plan. During the kickoff procedure, the detector trigger mode is set to TRIGLINK and the number of trigger points is configured. The detector is triggered by the synchronous trigger module and performs a measurement after receiving the trigger signal. A measurement occurs every time the trigger signal is detected, meaning that only one reading is recorded in the detector buffer at each energy setpoint position. The trajectory and trigger point parameters are transmitted to the motion controller and the synchronous trigger module, respectively. These trigger point parameters are stored in the synchronous trigger module’s sequence table in the form of relative positions. Before the scan, its internal position parameter is set to zero, ensuring alignment with the first row of the sequence table. Once these configurations are completed, a command is issued to the motion controller to begin the fly scan. The controller then synchronously drives the two monochromator motors according to the trajectory. Thereafter, the complete procedure listens for the scan end signal. On completion of the scan, the collect procedure gathers the data recorded in the detectors’ buffers.

For the soft fly scan Plan, data acquisition is accomplished through software based equal time interval collection while the motors move at a constant energy speed. In the kickoff procedure, the detector trigger mode is set to IMMEDIATE and the predefined trajectory is sent to the motion controller. Once the detector begins measuring, it takes readings as fast as its measurement configuration allows. After these configurations are complete, the controller drives the motors to move synchronously along the trajectory. In order to reduce the noise, multiple detector readings are acquired at uniform time intervals and their average is recorded in the Event.

#### 2.2.2. User interface

**Figure 4:**
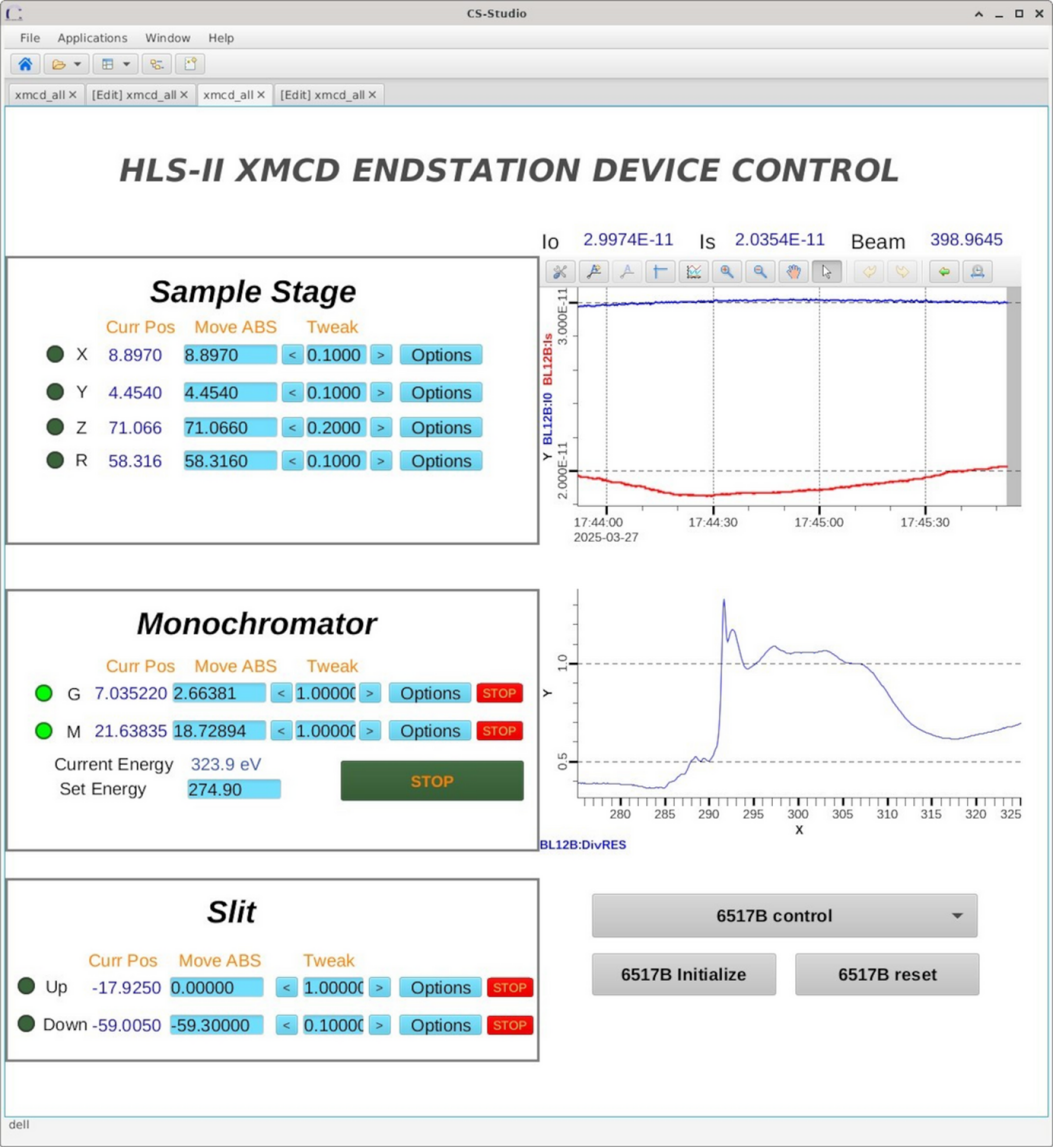
Device control GUI of the XMCD ECS.

The device control GUI was developed in *Phoebus*, as shown in Fig. 4 ▸. This GUI enables motion control of the slit, monochromator and sample stage motors, as well as real-time monitoring of the detectors’ data.

**Figure 5:**
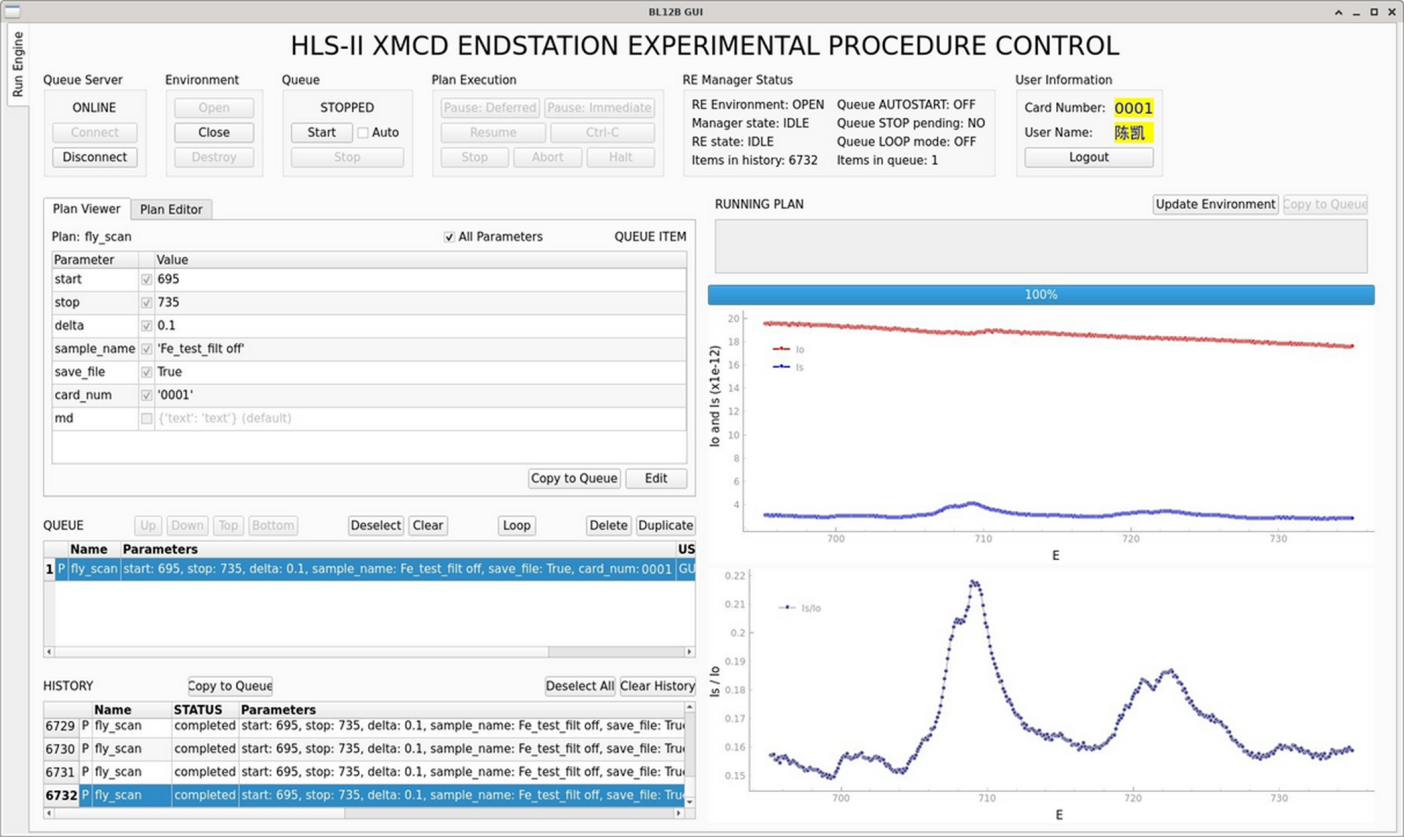
Experimental procedure control GUI of the XMCD ECS.

The experimental procedure control GUI refers to *Bluesky Widgets Demo* (NSLS-II, 2022 ▸) provided by NSLS-II and is developed with *PyQt5*. As shown in Fig. 5 ▸, this GUI is developed in a modular manner with the APIs and graphical widgets of *Queue Server*. The GUI is organized into three primary modules: the *Run Engine manager module* for managing the *Run Engine* environment of *Queue Server*, the *Plan queue manager module* for managing the Plan queue and the *live plot module* for real-time data visualization.

#### 2.2.3. Data acquisition

**Figure 6:**
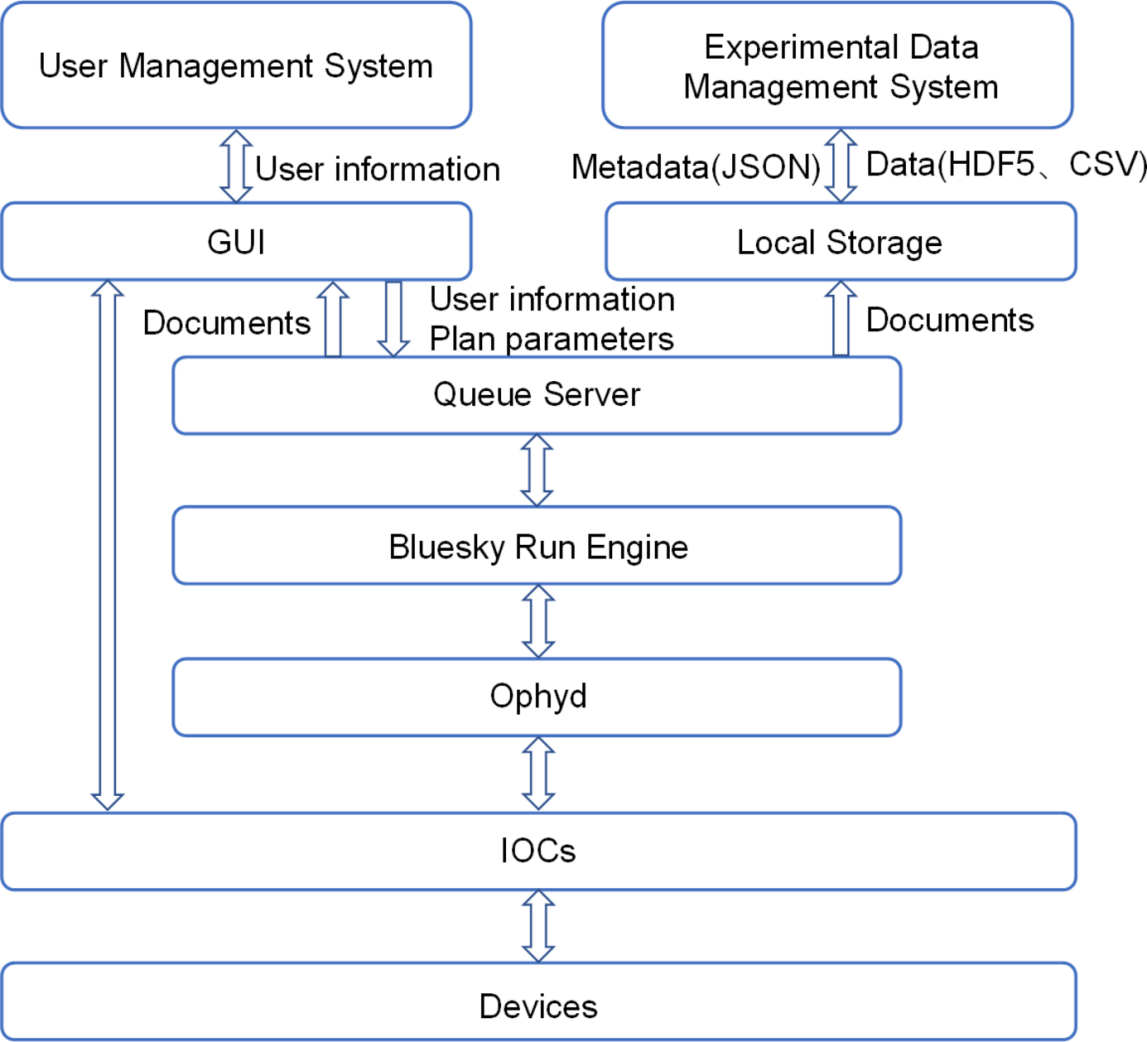
Data acquisition architecture of the XMCD ECS.

*Bluesky Event Model* organizes raw data and metadata into Documents which in Python are represented as dictionaries, including three primarily types: Start, Event and Stop. Start contains metadata such as user information, experimental parameters and sample information. Event is the raw data with associated timestamps. *Run Engine* passes instructions from Plan to the hardware through the hardware abstraction layer, and converts the collected experimental data into Documents. The data flow and control flow of the data acquisition architecture are shown in Fig. 6 ▸. During data acquisition, the Document stream generated by *Run Engine* is published to the data storage application callback function and the experimental procedure control GUI callback function through *ZMQ*.

**Figure 7:**
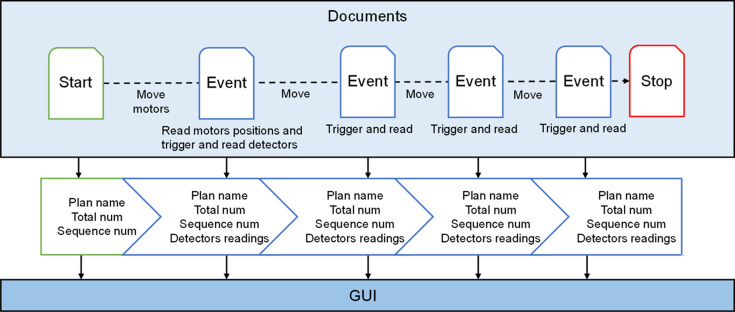
Data visualization Document stream processing.

The experiment procedure control GUI interacts with *Queue Server* to submit the Plan and receive the Document stream, as shown in Fig. 7 ▸. At the beginning of the Plan, the Plan name, total collection point number and sequence number are extracted from Start. Throughout the execution of the Plan, Events are parsed to retrieve the detector readings and the current sequence number which are then utilized to update the data plot and progress bar, thereby providing real-time feedback on the experiment procedure.

**Figure 8:**
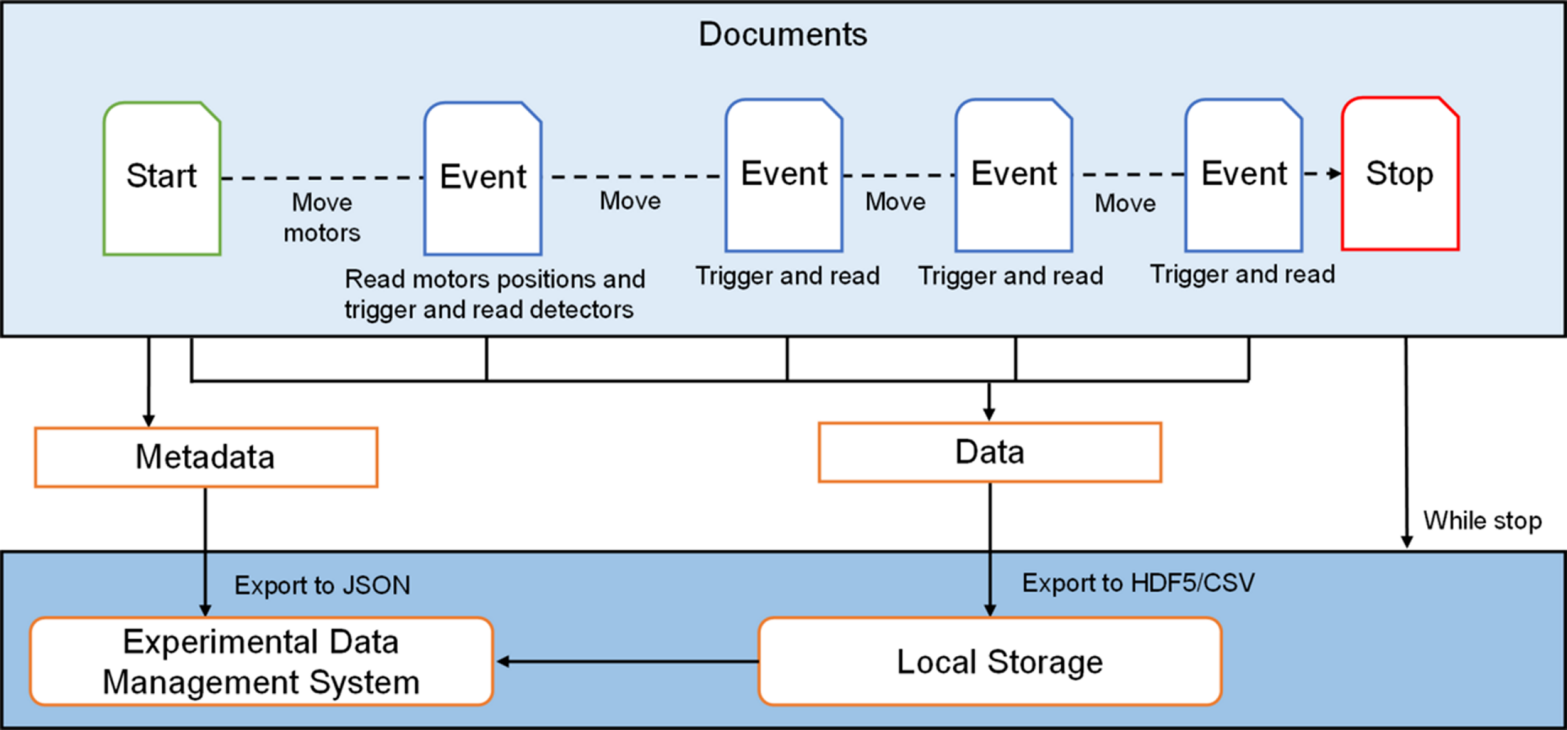
Data storage application Document stream processing.

To ensure proper authentication and data management, the user is required to input their card number and name. This information is used to retrieve user and proposal details from the user management system. A metadata request is sent to the experimental data management system, facilitating the construction of user information and roles. As shown in Fig. 8 ▸, the data storage application extracts metadata from Start and caches Events generated through the experiment. On receipt of Stop, the application initiates data export and storage. In accordance with the standards set by the experimental data management system and the beamline endstation, the metadata are exported in JSON format. A request is then sent to the experimental data management system. The raw data and metadata are exported in both HDF5 and CSV formats and stored in the local data server. To ensure data accessibility, the locally stored data are uploaded to the central data server in Rsync mode at the end of the Plan execution. This upload process includes file integrity verification and centralized storage, ensuring that users can access the data through web based services provided by the experimental data management system. This comprehensive data handling and storage process guarantees that all experimental data are accurately captured, securely stored and readily accessible for further analysis.

## 3. Simulation debugging environment

The XMCD ECS is designed with a simulation debugging environment to ensure efficient and reliable software development. This simulation debugging environment includes a virtual server platform, virtual devices and virtual IOCs. Virtual devices are developed based on the communication protocols of the beamline devices. On the virtual server platform, the virtual IOCs that communicate with the virtual devices use a specific real-time database. The records in this database are virtual records. The ECS software development and debugging can be carried out through these virtual records. When switching from simulation to the physical environment, these virtual records are replaced by the real records, enabling smooth online debugging of the control software in the physical environment.

### 3.1. Virtual server platform

The XMCD endstation employs *VMware* to construct a virtual server platform, and creates multiple virtual machines on it. These virtual machines serve various purposes, including file servers, web servers, development servers and soft IOCs. By utilizing server platform virtualization, multiple physical servers and network storage devices are integrated into a unified management platform. This approach integrates computing resources and storage capacity into a shared resource pool, enables dynamic scheduling and flexible allocation of resources, and greatly reduces hardware costs. High availability and fault tolerance guarantee uninterrupted operation of virtual servers during maintenance, upgrades or physical server failures. These technologies enhance the overall reliability of the system, ensuring that services remain unaffected by hardware issues.

### 3.2. Virtual device

**Figure 9:**
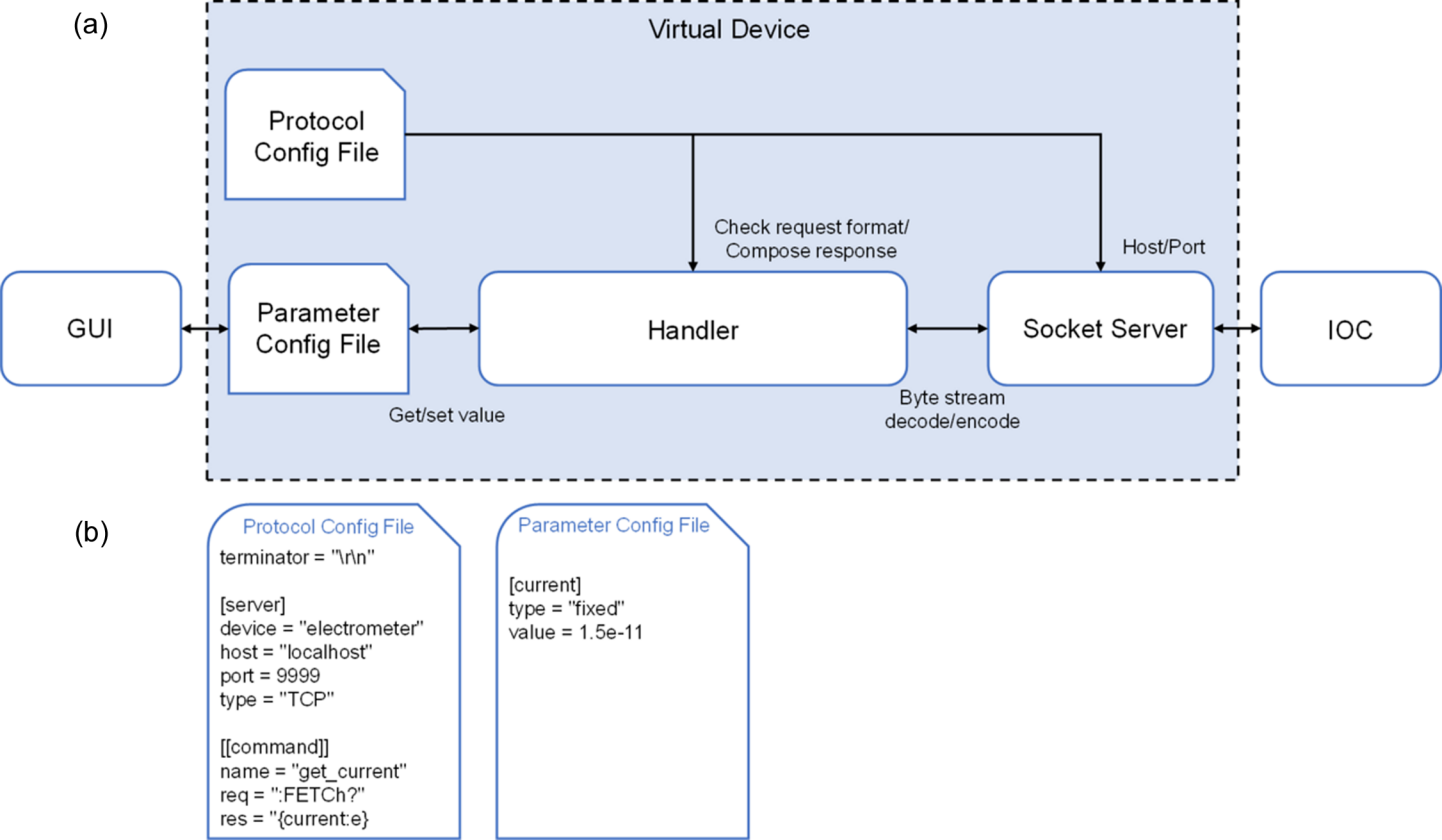
(*a*) Virtual device architecture. (*b*) Virtual device config flies.

Virtual devices are developed using *SocketServer* according to the communication protocols. *SocketServer*, a module in the Python standard library, offers server classes and request handler classes. The server class is responsible for managing communication tasks, while the request handler class handles protocol-related operations, such as interpreting incoming data, processing data and sending data back to the client. As shown in Fig. 9 ▸(*a*), the virtual device employs the server class to establish TCP/UDP communication services. The handler receives and interprets ASCII based byte stream. As shown in Fig. 9 ▸(*b*), config files are in TOML format and user-friendly virtual device configuration is achieved through a parameter config file and a protocol config file. The parameter config file contains the type and value of the virtual device parameters. The protocol config file includes the request and response formats associated with each parameter, the checksum and the terminator. Additionally, it includes communication details, such as the network transmission protocol type (TCP/UDP), host address and port number. By structuring the virtual device communication protocol in these config files, users can simulate various devices without developing code. *PyQt5* is used to build the GUI to facilitate real-time parameter management, such as parameter addition, deletion, modification and querying.

**Figure 10:**
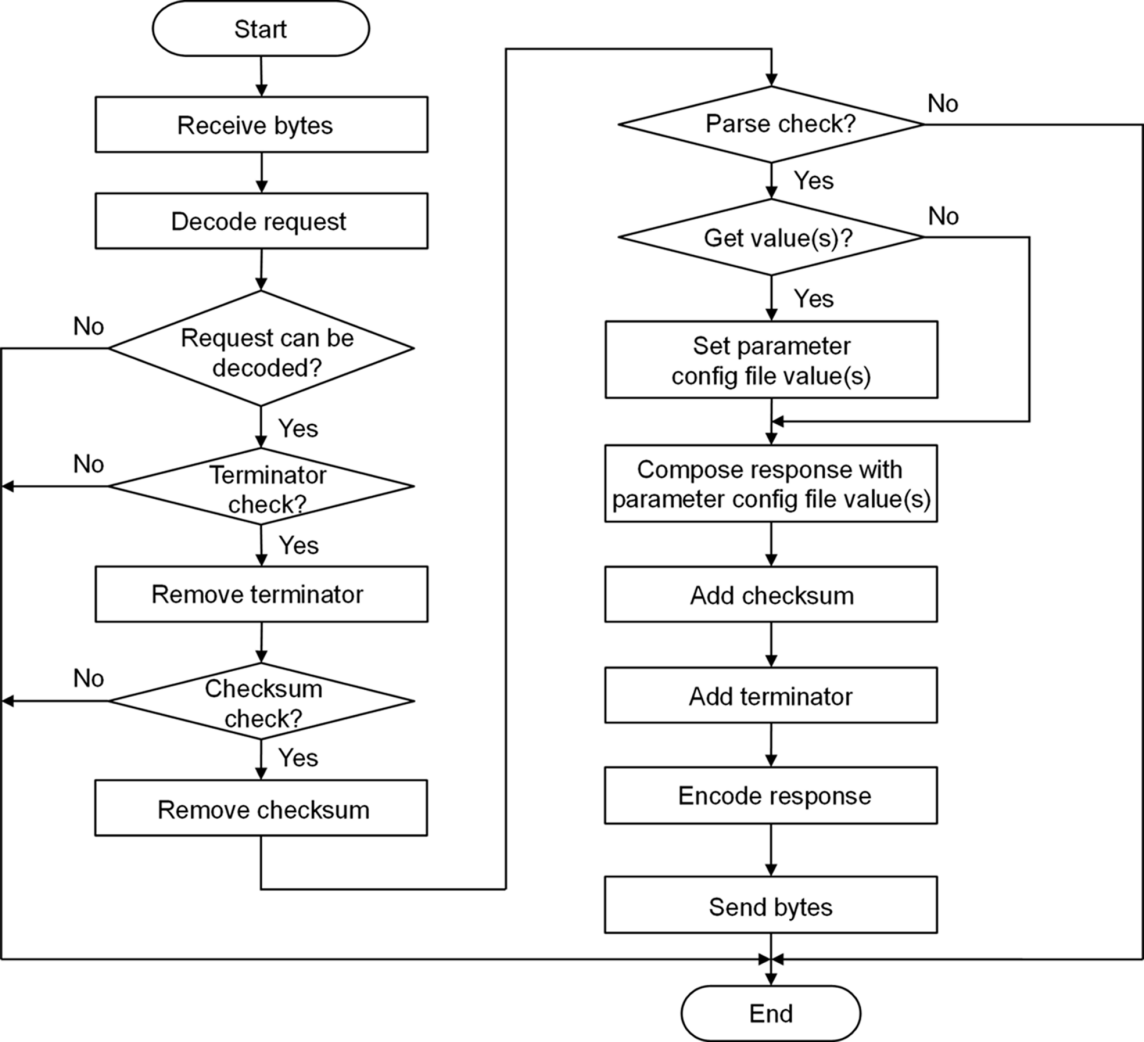
Virtual device byte stream decoding and encoding processes.

*StreamDevice* (PSI, 2018 ▸) is a general *EPICS* device support for devices with a ‘byte stream’ based communication interface. To implement the format check of the IOC request, a handler is developed with reference to the parameter format converters of *StreamDevice* and the Python string format() method. It can integrate parameter values with the response protocol format so that the response is returned to the IOC. Currently, this handler supports parameter data types, such as string, decimal integer, binary, octal, hex, scientific notation and fixed-point notation. Fig. 10 ▸ shows the virtual device byte stream decoding and encoding processes. After receiving the byte stream, the virtual device initiates the processing sequence by first decoding the request. It verifies the terminator and checksum of the request in turn. Once validated, the terminator and checksum are removed to facilitate further processing. Following this, a parsing check is performed. Regular expressions are used to extract both the parameter and protocol parts from the request. These extracted components are then cross-referenced against the request formats in the protocol config file. If a matching request format is identified, the parameter values derived from the parsing check are updated in the parameter configuration file. The corresponding response format and parameter values are combined into a response. After generating the response, the checksum and terminator are appended sequentially. The complete response is encoded and passed back to the IOC.

### 3.3. Simulation experiment results

**Figure 11:**
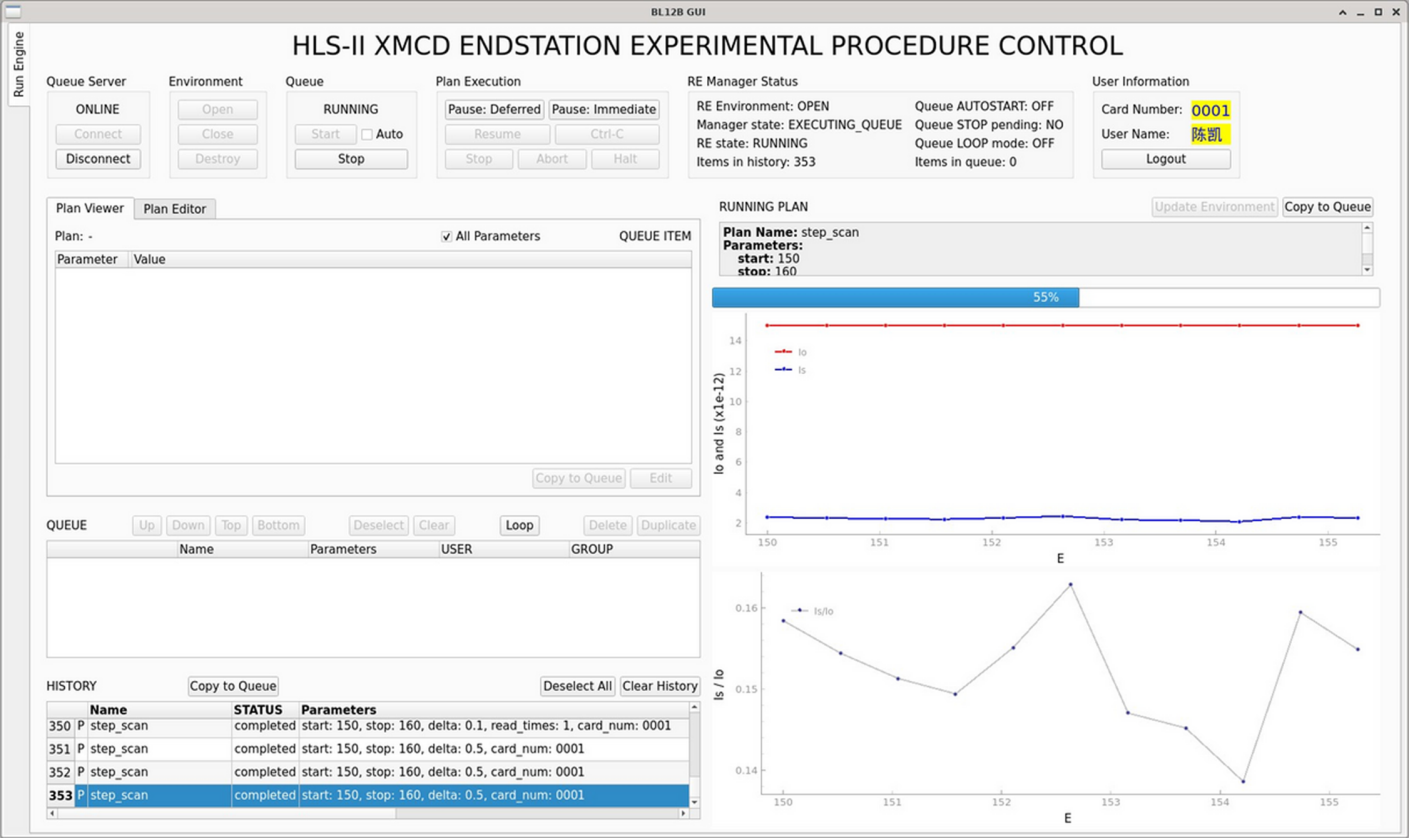
Simulation experiment GUI of the XMCD ECS.

The XMCD ECS with virtual IOCs for Keithley 6517B electrometers is deployed on a Debian 12 virtual machine hosted on a virtual server platform. Virtual electrometers are created according to the Keithley 6517B communication protocol. Two virtual monochromator motors, energy-position conversion signal and position-energy conversion signal are established based on *Ophyd*. The offline simulation step scan experiment results prove the functionality of the ECS, as shown in Fig. 11 ▸. Key features verified during testing include user login management, *Queue Server* management, Plan queue editing, Plan execution, real-time data visualization, local data storage and data synchronization. It confirms that this system is ready for operation and can effectively manage the experimental procedure.

## 4. Results

**Figure 12:**
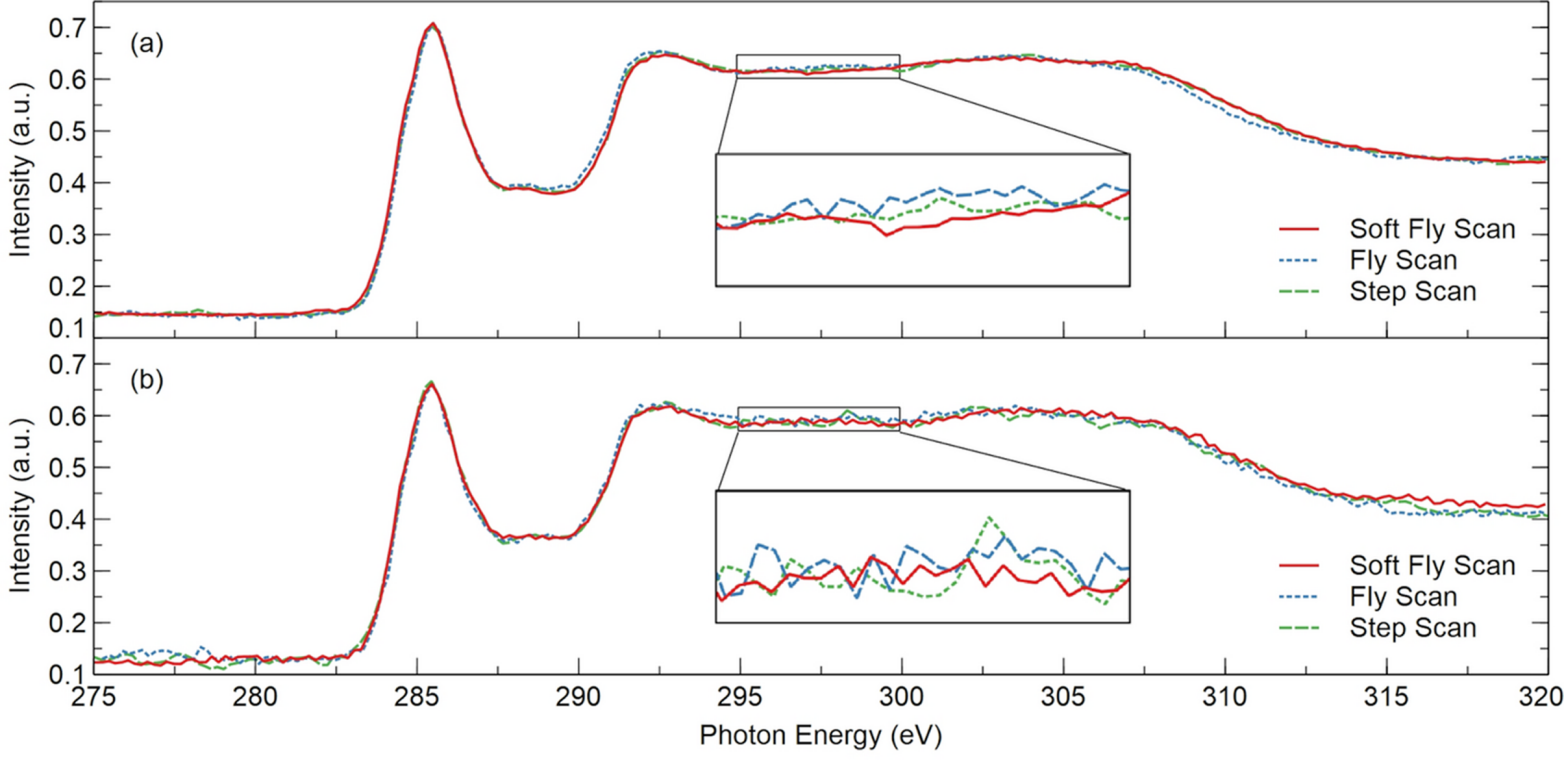
(*a*) XAS spectra of the C *K*-edge measured with three different modes: soft fly scan, fly scan, step scan. (*b*) XAS spectra of the C *K*-edge measured with three different modes: soft fly scan, fly scan, step scan. The incident photon beam intensity is reduced by 55%.

The XAS measurements of graphite specimens were performed at the XMCD endstation using TEY detection mode. A systematic energy scan spanning 275–320 eV was implemented with a step of 0.2 eV. For both the fly scan and the soft fly scan, the detector integration time was 20 ms. If the same integration time is applied to the step scan, the results will exhibit the lowest accuracy due to the stop–start nature of the step scan and that the monochromator alignment in the stopped state is less accurate than while in motion (Hidas *et al.*, 2022 ▸). The primary time consumption in step scan arises from motor dead time. To enhance the signal-to-noise ratio (SNR), the detector integration time for step scan was extended to 200 ms, and a digital averaging filter with 20 readings was applied. The experimental results are presented in Fig. 12 ▸(*a*). The step scan required 54 min for spectrum acquisition, while the fly scan and soft fly scan each took 6 min. The spectra represent the sample signal *I*_s_ normalized by the gold mesh signal *I*_0_. In all three results, the highest peak falls within the same 0.2 eV wide acquire window corresponding to 285.4 eV, and the full width at half-maximum (FWHM) of the peak is 2.6 eV. The peak shape and position exhibit consistency across all three results. In an XAS spectrum, when the incident X-ray energy matches the binding energy of an electron within the sample, the number of X-rays absorbed by the sample increases significantly, resulting in a sharp decrease in transmitted X-ray intensity. This phenomenon produces an absorption edge. Although the absorption edge is considered a signal feature rather than noise, it substantially increases the range of data fluctuations. Therefore, selecting a flat region without the absorption edge is more suitable for obtaining an SNR that accurately reflects the background noise level. To evaluate the data quality, the linear least squares regression method is employed to fit the data from 295 to 300 eV and the root mean square error (RMSE) is calculated. A smaller RMSE corresponds to a higher SNR. The RMSEs for soft fly scan, fly scan and step scan are 2.334 × 10^−3^, 2.927 × 10^−3^ and 2.13 × 10^−3^, respectively. Soft fly scan and fly scan achieves a tenfold reduction in measurement duration while maintaining SNR levels comparable to step scan. By adjusting the slit, the intensity of the incident photon beam was reduced by 55%. Utilizing the same parameters, additional experiments were conducted under low light intensity conditions, with the results displayed in Fig. 12 ▸(*b*). The RMSEs for soft fly scan, fly scan and step scan are 4.386 × 10^−3^, 6.402 × 10^−3^ and 7.577 × 10^−3^, respectively. By implementing noise-reduction methods, the soft fly scan achieves a superior SNR in the spectrum compared with the step scan and fly scan. Soft fly scan not only enhances experimental efficiency by addressing the slow speed of step scan but also maintains reliable experimental quality even under conditions with a poor SNR. This method significantly reduces the exposure time of the sample to the photon beam, minimizing potential damage to delicate samples.

**Figure 13:**
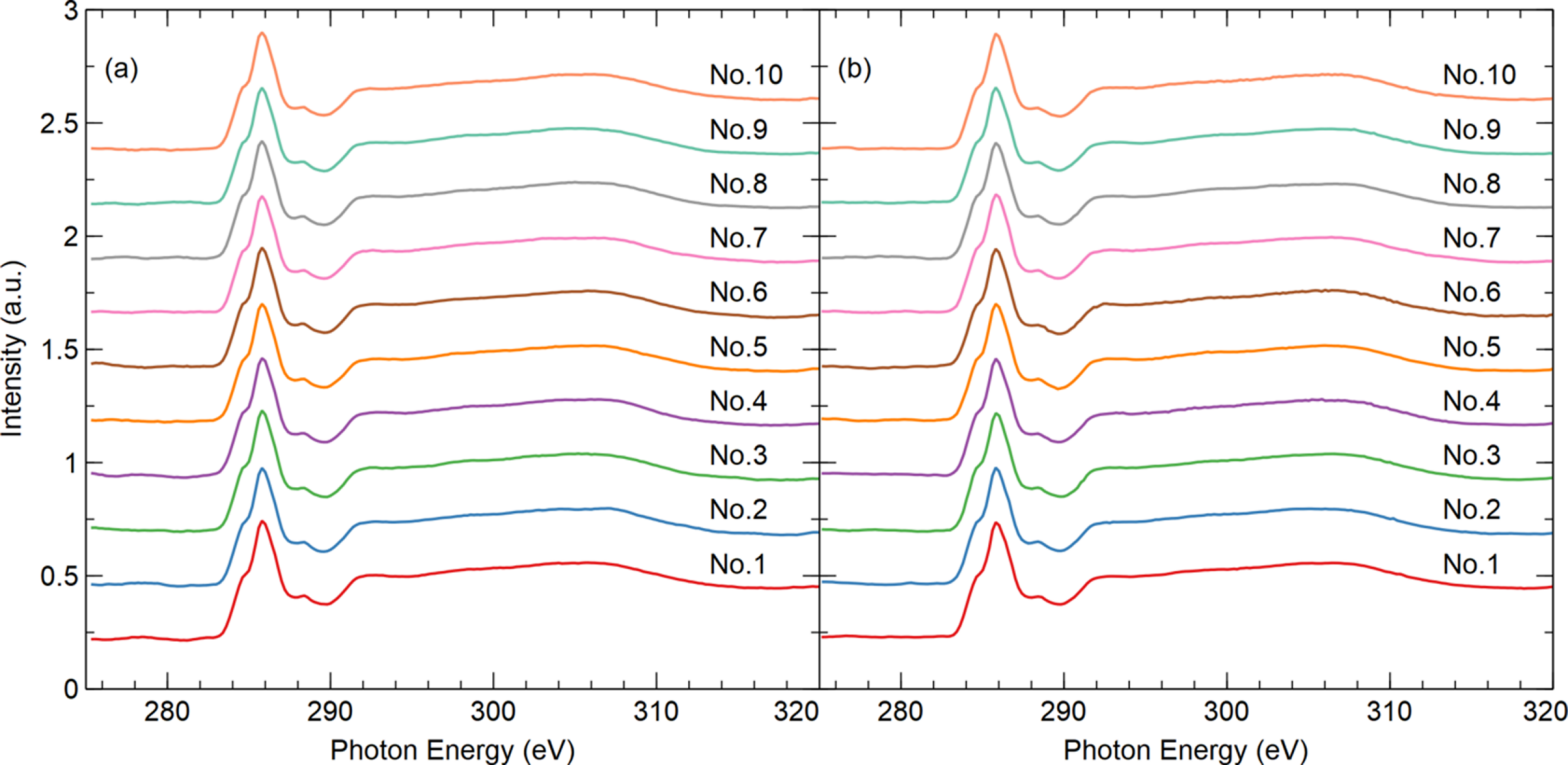
(*a*) XAS spectra of the C *K*-edge measured 10 times using fly scan. (*b*) XAS spectra of the C *K*-edge measured 10 times using soft fly scan. Spectra are vertically shifted for easier observation.

To verify the repeatability of the measurement results, 10 datasets were collected from a carbon sample using fly scan and soft fly scan. The energy range was set from 275 to 320 eV with a resolution of 0.2 eV. For easier observation, the original spectra are vertically shifted, and the experimental results are displayed in Fig. 13 ▸. The RMSE of each result compared with the average of 10 results is shown in Table 1 ▸. The average RMSEs for fly scan and soft fly scan are 2.858 × 10^−3^ and 2.875 × 10^−3^, respectively. Both values approximate zero, indicating that under consistent experimental conditions the results of multiple experiments using fly scan and soft fly scan have high repeatability and stability.

## 5. Discussion and conclusions

The HLS-II XMCD endstation has developed an ECS based on *Bluesky* and *EPICS*, which unifies the abstraction of motors, detectors and other devices required for the experiment. This system also standardizes a variety of experimental procedures, including step scan, fly scan and soft fly scan. The GUI provided has experimental procedure control and real-time data display functions. The experimental raw data and metadata are stored locally in a standard data format and automatically synchronized to the experimental data management system. The Plan queue service allows users to schedule the order of multiple scan tasks and execute them automatically. The control architecture incorporates a design that separates control flow and data flow, ensuring the stability and reliability of the experimental procedure. The experimental results demonstrate that soft fly scan reduces the scan completion time by nearly an order of magnitude while maintaining a high SNR, significantly enhancing both experimental efficiency and data quality for the user. This ECS architecture will be implemented at the beamline endstations of the HLS-II and the Hefei Advanced Light Source (HALF) to establish a unified control system architecture for spectroscopy, imaging and diffraction experiments and reduce software development and maintenance costs. It has been promoted and applied to the HLS-II Spectral Radiation Standard and Metrology beamline endstation, ensuring a unified and efficient approach across multiple beamlines. By leveraging server platform virtualization and device virtualization technologies, a simulation debugging environment is established. This environment can carry out simulation experiments and functional testing of the ECS during endstation construction, ensuring seamless integration with the actual beamline endstation. The simulation debugging environment provides support for automated deployment of ECSs.

## Figures and Tables

**Table 1:** RMSE of repeatability test for fly scan and soft fly scan

Experiment No.	Fly scan RMSE (10^−3^)	Soft fly scan RMSE (10^−3^)
1	3.165	2.877
2	3.273	2.517
3	2.73	3.922
4	2.926	2.293
5	1.728	2.031
6	3.2	3.345
7	2.56	3.059
8	2.622	2.751
9	3.459	3.056
10	2.92	2.903
Average	2.858	2.875
